# Catastrophic health expenditure and impoverishment in households of persons with depression: a cross-sectional, comparative study in rural Ethiopia

**DOI:** 10.1186/s12889-019-7239-6

**Published:** 2019-07-11

**Authors:** Yohannes Hailemichael, Charlotte Hanlon, Kebede Tirfessa, Sumaiyah Docrat, Atalay Alem, Girmay Medhin, Crick Lund, Dan Chisholm, Abebaw Fekadu, Damen Hailemariam

**Affiliations:** 1 Department of Reproductive Health and Health Services Management, School of Public Health, College of Health Sciences, Addis Ababa University, Addis Ababa, Ethiopia; 2 Department of Health Economics, Policy and Management, College of Health Sciences, Jimma University, Jimma, Ethiopia; 3 Department of Psychiatry, School of Medicine, College of Health Sciences, Addis Ababa University, Addis Ababa, Ethiopia; 4 Centre for Global Mental Health, Health Service and Population Research Department, Institute of Psychiatry, Psychology and Neuroscience, King’s College, London, UK; 5 Centre for Innovative Drug Development and Therapeutic Trials for Africa (CDT-Africa), College of Health Sciences, Ababa University, Addis Ababa, Ethiopia; 6 Alan J. Flisher Centre for Public Mental Health, Department of Psychiatry and Mental Health, University of Cape Town, Cape Town, South Africa; 7 Aklilu-Lemma Institute of Pathobiology, Addis Ababa University, Addis Ababa, Ethiopia; 8 Department of Mental Health and Substance Abuse, World Health Organization, Geneva, Switzerland; 9 Department of Global Health & Infection, Brighton and Sussex Medical School, Brighton, UK; 10 Centre for Affective Disorders, Department of Psychological Medicine, Institute of Psychiatry, Psychology and Neuroscience, King’s College, London, UK

**Keywords:** Depression, Disability, Catastrophic health expenditure, Impoverishment, Low-and middle-income country, Universal health coverage

## Abstract

**Background:**

The extent of catastrophic health expenditure and impoverishment associated with depression in low-and middle-income countries is not known. The aim of this study was to estimate the incidence and intensity of catastrophic out-of-pocket (OOP) health expenditure, level of impoverishment and coping strategies used by households of persons with and without depression in a rural Ethiopian district.

**Methods:**

A comparative cross-sectional survey was conducted, including 128 households of persons with depression and 129 households without. Depression screening was conducted using the Patient Health Questionnaire, nine item version (PHQ-9). People in the depression group were classified into high and low disability groups based on the median value on the World Health Organization Disability Assessment Schedule (WHODAS) polytomous summary score.

Health expenditure greater than thresholds of 10 and 25% of total household consumption was used for the primary analyses. The poverty headcount, poverty gap and normalized poverty gap were estimated using retrospective recall of total household expenditure pre- and post-OOP payments for health care. Linear probability model using binreg command in STATA with rr option was used to estimate risk ratio for the occurrence of outcomes among households with and without depression based on level of disability.

**Results:**

Catastrophic OOP payments at any threshold level for households with depression and high disability were higher than control households. At the 10% threshold level, 24.0% of households of persons with depression and high disability faced catastrophic payments compared with 15.3% for depression and low disability and 12.1% for control households (*p* = 0.041). Depression and high disability level was an independent predictor of catastrophic OOP payments: RR 2.1; 95% CI:1.1, 4.6.

An estimated 5.8% of households of persons with depression and high disability were pushed into poverty because of paying for health care compared with 3.5% for households of persons with depression and low disability and 2.3% for control households (*p* = 0.039).

**Conclusions:**

Households of people with depression and high disability were more likely to face catastrophic expenditures and impoverishment from OOP payments. Financial protection interventions through prepayment schemes, exemptions and fee waiver strategies need to target households of persons with depression.

**Electronic supplementary material:**

The online version of this article (10.1186/s12889-019-7239-6) contains supplementary material, which is available to authorized users.

## Background

According to the World Health Organization (WHO), out-of-pocket (OOP) payments for health care at the point of service are an inequitable means of financing a health system [1]*.* Nevertheless, globally, OOP payments contribute a significant and increasing share of current health spending [2]. On average, OOP payments for people in low- and middle-income countries (LMICs) represent around 40% of health spending and present a significant barrier to access and utilization of health services [2].

Although health care financing reform in Ethiopia has been in process since 1998, OOP payments remain a significant financial burden on households [3]. User fees are waived for poor people as a means of financial protection; however, the implementation of this programme is weak [4]. Furthermore, few households are eligible for a waiver as there is a quota for each village (*kebele*) leading to limited benefit for most poor households. As a result, households often resort to borrowing money from family, friends, money-lenders, or to selling their assets and reducing consumption [5, 6].

There is compelling evidence that OOP payments act as sources of catastrophic expenditures and impoverishment [7]. Catastrophic OOP payments occur when spending on health care is above a certain threshold that results in financial distress [8]. However, the threshold levels for estimating catastrophic payments differ in the literature, ranging from 10% [9] to 25% of total consumption expenditure [10]. In a study on catastrophic health payments from 2010,the global incidence of catastrophic spending at the 10% threshold was estimated to be 11·7% [10].

Nonetheless, even small amounts of health expenditure may result in catastrophe and impoverishment for vulnerable households [11, 12]. Impoverishment occurs when a household that is above the poverty line pre-payment crosses the poverty line after paying (post-payment) for health care, shifting from non-poor to poor [13]. In addition, some households who were poor become even poorer after paying for health care [14]. A recent global analysis of impoverishment in 122 countries reported that 1.4% of the world’s population were impoverished by OOP payments on health care at the $1·90 per day poverty line [15].

Households that include a member with depression face disproportionately high catastrophic costs because of OOP payments [16]. In India, the incidence of catastrophic OOP payments for depression in women was 14.6% compared to 4.9% in the control group [16]. In Pakistan, OOP payments for the treatment of depressive disorder also led to significant costs for households [17]. In the limited studies from Ethiopia, depression has been found to be associated with increased use of healthcare, which may result in increased costs [18, 19]. While these studies contribute to the knowledge base of the impact of depression, they have not considered the magnitude of household catastrophic health expenditures and impoverishment associated with depression because of health care use. Moreover, many studies rely on self-report symptom scales rather than clinical diagnoses to define depression and do not consider the associated level of disability [20–23]. International diagnostic criteria include disability as a key criterion for diagnosis of depression [24, 25], and the World Health Organization clinical guidelines for depression require the presence of disability to define ‘moderate-severe depression’ which would require a clinical intervention [26]. Therefore, disability could be a key factor affecting health care costs in a person with depression, but the impact of disability has seldom been investigated to date.

The study reported in this paper is part of the multi-country Emerald programme (Emerging mental health systems in low- and middle-income countries) which sought to provide rigorous, population-based evidence about the adequacy and fairness of mental healthcare financing [27]. The objective of this sub-study was to estimate catastrophic OOP healthcare payments, level of impoverishment and financial coping strategies adopted by households of persons with depression of differing levels of disability compared to control households without an index person with depression.

## Methods

### Study design, setting and participants

A population-based, comparative, cross-sectional study was conducted in 2015 in Sodo district of the Southern Nations, Nationalities and Peoples’ Regional state of Ethiopia. Sodo district had an estimated population of 161,952 in 2012, of whom almost 90% reside in rural areas [28].

Sodo district is the setting for an implementation research project (the Programme for Improving Mental health carE; PRIME) which is evaluating the impact of integrating care for priority mental disorders into primary care [29]. Sodo district was selected by PRIME because it was mostly rural (in keeping with the rest of Ethiopia), included both highland and lowland topography, was accessible from Addis Ababa and neighboured a district where a psychiatric nurse-led out-patient unit was available [30]. As part of PRIME, primary health care workers in the eight health centres in Sodo district were trained to detect and treat depression, severe mental disorders (psychosis and bipolar disorder), epilepsy and alcohol use disorders [30]. The Emerald project was linked to PRIME with the purpose of investigating what health system strengthening was needed to support integrated primary mental health care at the district level.

The sample of people with depression was recruited from consecutive people attending the health centers who were identified by primary care staff as having a probable diagnosis of depression or who screened positive on the Patient Health Questionnaire, nine item version (PHQ-9) at the baseline of PRIME and were thought to require treatment. The PHQ-9 was originally used to identify probable depression in primary care samples in the United States of America [31]. The PHQ-9 has been validated in Ethiopia in a hospital out-patient clinic [32] and in primary care attendees in health centers in a district neighboring the location of the current study [19].

The control sample was recruited through Emerald and included people who attended the health centers on the same day as the person with depression but who did not have a primary care worker diagnosis of depression and who had a PHQ score < 5, matched with participants in the depression group by gender, age (±5 years) and *gott* (lowest residential administrative area). A household socio-economic survey for the case and the control households was conducted by Emerald within two to four weeks of primary care attendance.

The sample size was estimated to detect a difference in catastrophic OOP health payments, assuming the proportions of households with catastrophic expenditures were 14.6% for households that have members with depression and 4.9% for those that do not [19]. With the assumption of 95% confidence in the estimate, a power of 80%, household ratio of 1:1 and detectable risk ratio of 2.97, the resulting minimum sample size was 294 (147 households with depression and 147 control households).

#### Data collection

Data collection was conducted from March to August 2015 by trained data collectors using an adapted and abbreviated version of the World Health Organization Study on global Ageing and adult health (SAGE) survey instrument [33]. The questions related to this paper included the following: basic demographic and socio-economic characteristics (household head, age, sex, education, residential location, household size and income), household consumption expenditure for differing time periods, OOP health payments and coping strategies used for financial difficulties.

### Variables

The primary outcome in this study was catastrophic OOP health payments, and the secondary outcomes were impoverishment and financial coping mechanisms adopted by the household. The main explanatory variable was depression status of the index person (household containing a person with or without depression). In addition, household size, gender and educational status of the head of the household, presence of older people (> 60 years old), presence of children younger than 15 years in the household, place of residence (urban or rural) and disability level of the index person with depression were considered as covariates.

### Outcome measures

#### Incidence and intensity of catastrophic health payments

According to O’Donnell (2008), the incidence of catastrophic payments can be estimated from the fraction of a sample with health care costs as a share of total (or non-food) expenditure exceeding the chosen threshold [34]. However, there is no uniformly accepted threshold for estimating the incidence of catastrophic OOP payments. In the literature, the threshold at which health payments become catastrophic varies between 5 and 40% of the household income or consumption expenditure [8, 11, 14]. In this study, threshold values of 10 and 25% of total consumption were used, as proposed by the World Health Organization for monitoring financial protection for universal health coverage (UHC) [10].

Incidence (headcount) of catastrophic OOP estimates does not give information on the extent (intensity) of catastrophic costs (by how much household OOP payments exceed the catastrophic threshold). Hence, intensity is estimated through catastrophic overshoot and mean positive overshoot (MPO) [34, 35]. Catastrophic overshoot is defined as the mean value by which household out-of-pocket expenditure on the illness, as a percentage of total household expenditure, exceeded the 10 and 25% threshold used to define catastrophic household expenditure. Mean positive overshoot is defined as the mean level by which out-of-pocket expenditure on the illness, by a household reporting catastrophic health expenditure, exceeded the 10 and 25% threshold used to define catastrophic household expenditure [35].

OOP payments were measured in terms of expenditures related to consultation, examination and diagnostic tests, medications, services provided by traditional healers, and payments for other health-related services in the past 30 days. OOP payments made for hospital care were measured for the last 12 months. OOP payments were estimated in terms of Ethiopian Birr and converted to US dollars (US$). The average 2015 exchange rate of 20.69 Ethiopian Birr (ETB) to US$1 was used [36].

Household consumption expenditure (i.e. consumption of food produced by the household or purchased in the market or given in kind to the household, consumption of non-food items for daily use, consumption of consumer durables, consumption of health care goods, consumption related to transfers out to household or community) was estimated in the last 30 days before the survey. The estimated consumption expenditure for each household was adjusted for household size and age using the Organization of Economic Co-operation and Development (OECD) modified scale to obtain an adult equivalent score [37]. The resulting household consumption expenditure was then classified into five consumption quintiles.

#### Out-of-pocket payments and impoverishment

OOP payments can be impoverishing when a household’s level of expenditure before making health payments (pre-payment) was above the poverty line, but then fell below the poverty line after health expenditures (post-payment) [14]. In this study, both pre- and post-payment poverty status were ascertained cross-sectionally, with retrospective report. We measured whether the effect of OOP expenditures could lead to poverty using three measures, as recommended by Wagstaff and van Doorslaer [14]: (i) poverty head count, which is the proportion of households living below the poverty line, (ii) poverty gap, or the average amount by which resources fall short of the poverty line, and (iii) normalized poverty gap, obtained by dividing the poverty gap by the poverty line (i.e. for international comparison). Therefore, measures of poverty impact (PI) of OOP health payments are the difference between the relevant pre-payment and post-payment measures for the headcount, the poverty gap and the normalized poverty gap.

Estimating these three measures requires setting a poverty line and assessing the extent to which OOP payments push households below it. The poverty line was defined using the Ethiopian national poverty line of total consumption expenditure of US$ 182.74 per adult equivalent per year in 2012 [38]. This figure was used to estimate the poverty headcount, the gap and the normalized gap before and after healthcare payments.

#### Coping strategies for financial difficulties

Both incidence and intensity of catastrophic out-of-pocket payment thresholds ignore coping strategies implemented for financial difficulties by the household. The type of coping mechanism employed can have important implications for the recovery of the household from poverty in the short- and long-term. Therefore, we analyzed financial coping mechanisms that included selling of assets, savings, borrowing, consumption reduction, withdrawal of children from school, interruption of medical visits and taking on extra work.

#### Disability measures

Disability was assessed using the 12-item fully structured interviewer administered version of the World Health Organization Disability Assessment Schedule second version (WHODAS–II) [39]. WHODAS–II asks about difficulties faced in the last 30 days due to health conditions, such as getting dressed, joining in community activities and day-day work, with responses ranging from none to extreme/cannot do; it also asks about the number of days lost from work in the past 30 days due to health conditions. The Amharic version of this instrument was validated for people with major depression in Ethiopia [18]. For this study, people enrolled in the depression cohorts were classified into two groups based on the median of the WHODAS-II polytomous summary score (possible score range: 0–100), with the hypothesis that greater disability would be associated with incurring higher catastrophic OOP payments and impoverishment. Hence, the following classification was made: (A) Depression and high disability (WHODAS- II score ≥ 33.3) and (B) Depression and low disability (WHODAS-II score < 33.3).

#### Statistical analysis

Data management was performed using Epidata for Windows, Version 3.1 [40] and statistical analysis was performed using Stata Version 13.1 [41]. Preliminary analysis of all variables was conducted using descriptive analysis. We analyzed differences between socio-economic characteristics, OOP health payments and financial coping strategies using one-way analysis of variance, the non-parametric Kruskal-Wallis test and chi-square test (χ^2^) for statistical significance. The chi-square test was also used to examine the unadjusted association of depression status with catastrophic payments at the 10 and 25% thresholds. The risk of encountering catastrophic out-of-pocket payments and potential explanatory variables were modeled using linear probability model employing binreg command in STATA with rr option. The strength of these associations was quantified using risk ratio (RR) with corresponding 95% confidence intervals.

Coping mechanisms adopted by the households were analyzed by comparing households of persons with disability and depression and households without depression. Linear probability models using binreg command in STATA with rr option were fitted to examine the independent effect of depression and level of disability on the risk of encountering catastrophic expenditure and a greater likelihood of implementing financial coping strategies**.** The models were run separately for the different coping strategies using the same set of independent variables. Risk ratio with corresponding 95% confidence intervals were used to quantify the effect size of the associations.

## Results

A total of 129 households of a person with depression and 129 comparison households were recruited. Of these, one household of a person with depression was excluded due to missing data on household consumption expenditure, income and OOP payments for health care. In the analysis, 128 households with depression and 129 households without depression were included.

### Household characteristics

Household socio-demographic, economic and selected health outcome variables of the 257 participants are presented in Table 1. The percentage of household heads with no formal education in the three groups did not differ significantly (*p* = 0.164). The mean age of the head of the household was higher in households of persons with depression and high disability (*p* = 0.046).

**Table 1:** Characteristics of study households by mental health condition and severity

Household (HH) Characteristics	Households of person with depression (*n* = 128)		Comparison households (*n* = 129)
	Depression and high disability (*n* = 65)	Depression and low disability (*n* = 63)	
Socio-demographic and economic			
Age of HH head (years), mean (SD)	**48.7 (11.8)**	44.1 (12.9)	44.2 (13.8)
HH size, mean (SD)	5.2 (2.1)	4.9 (1.9)	5.0 (2.0)
Adult Equivalent Size, mean (SD)	2.7 (0.9)	2.6 (0.8)	2.6 (0.8)
HH with at least one older person ≥60 years old, n (%)	15 (23.0)	14 (22.9)	28 (21.8)
HH with at least one child younger than 15 years, n (%)	56 (86.1)	49 (80.3)	112 (87.5)
Residence, n (%)			
Rural	46 (70.8)	57 (90.5)	103 (79.8)
Urban	19 (29.2)	6 (9.5)	26 (20.2)
Gender, n (%)			
Male	52 (80.0)	49 (80.3)	96 (75.0)
Female	13 (20.0)	12 (19.7)	32 (25.0)
HH Head marital status, n (%)			
Never married	1 (1.5)	3 (5.0)	6 (4.7)
Married	52 (80.0)	50 (83.3)	98 (76.6)
Separated/divorced/widowed	12 (18.5)	7 (11.7)	24 (18.7)
HH Head education, n (%)			
No formal education, n (%)	44 (67.67)	38 (60.3)	67 (52.4)
Primary education	13 (20.0)	19 (30.2)	36 (28.1)
More than primary	8 (12.3)	6 (9.5)	25 (19.5)
HH with health insurance, n (%)	2 (3.1)	1 (1.6)	9 (6.9)
Annual total consumption, median (IQR) ††$	**369.7 (253.8, 519.0)**	485.9 (320.4, 795.5)	495.6 (339.6, 778.3)
Annual health payments, mean (SD) ††$	**45.3 (111.4)**	37.6 (50.7)	28.9 (39.2)
Clinical Characteristics			
Functioning			
Index patient WHODAS, median (IQR)	**47.2 (38.9, 61.1)**	16.6 (11.1, 25.0)	–
Symptom scores			
Index patient PHQ-9, median (IQR)	**12.0 (9.0, 15.0)**	9.0 (6.0, 11.0)	–

Bold, significant at P < 0.05 using Pearson’s χ^2^.for categorical data; Kruskal-Wallis for non-normal continuous data; Analysis of variance for normal data; **††** adult equivalent; $ = USD; USD 1 = Birr 20.69 (2015); HH (household);

WHODAS (World Health Organization Disability Assessment Scale); IQR (Inter-Quartile Range); SD (Standard Deviation); PHQ-9 (Patient Health Questionnaire − 9-item version)

Median annual consumption was significantly lower in households with depression and high disability (US$ 369.7) compared with households with depression and low disability (US$ 485.9) and households without depression (US$ 495.6), *p* = 0.007.There was no significant difference between the percentage of households enrolled in any form of health insurance: 1.6% in households of a person with depression and 6.9% in comparison households.

Mean household OOP payments on healthcare were highest in households with depression and high disability (US$45.3) compared with households with depression and low disability (US$37.6) and households without depression (US$28.9) (*p* < 0.001).

In Additional file 1: Table S1, the household budget share of food, non-food and health care payments is presented. An average of 74.9% (95%CI:70.7,79.2) of total household consumption was spent on food by households of persons with depression and high disability compared with 76.5% (95%CI: 72.7, 80.4) in households with depression and low disability and 78.7% (95%CI: 75.9, 81.4) in households without depression, which was non-significant (*p* = 0.201). A comparison of the share of annual OOP health payments relative to annual consumption was 7.5% (95%CI: 4.3,10.7) for households of persons with depression and high disability, 6.7% (95%CI: 4.4, 9.0) for households of persons with depression and low disability and 4.9% (95%CI:3.7,6.2) for households without a person with depression (*p* = 0.230).

### Incidence and intensity of catastrophic health payments

The sensitivity analysis for the incidence and intensity of catastrophic health payments is reported in Table 2. There was an inverse relationship between catastrophic incidence (percentage of households experiencing catastrophic OOP health expenditure) and the various thresholds. For example, 24.0% of households of persons with depression and high disability, 15.3% of households containing depression and low disability, and 12.1% of households without persons with depression reported total OOP payments exceeding 10% of total consumption expenditure (*p* = 0.041). Increasing the threshold to 25% reduced the catastrophic incidence to 5.7% in households of person with depression and high disability,5.5% in households of persons with depression and low disability and 1.2% in households without depression (*p* = 0.284).

**Table 2:** Sensitivity analysis of catastrophic out-of-pocket healthcare payments at various threshold levels

Mental health conditions	Catastrophic healthcare expenditure measures as a share of total consumption	Threshold level			
		5%	10%	15%	25%
Depression and high disability	Headcount (%)	46.1	**24.0**	11.5	5.7
	Overshoot (%)	4.0	2.6	2.0	1.2
	Mean positive overshoot (%)	8.8	**17.3**	17.5	21.5
Depression and low disability	Headcount (%)	38.8	15.3	9.2	5.5
	Overshoot (%)	3.5	1.9	1.2	0.5
	Mean positive overshoot (%)	8.1	9.1	10.4	13.9
Comparison without depression	Headcount (%)	32.9	12.1	6.0	2.4
	Overshoot (%)	1.9	0.9	0.4	0.02
	Mean positive overshoot (%)	5.9	7.2	7.6	8.7

Bold, significant at P < 0.05 for Pearson’s χ^2^ comparing catastrophic head count by depression sub-groups

The intensity of catastrophic out-of-pocket payments at all threshold levels was also larger for households of persons with depression and high disability compared to households with depression and low disability and households without a person with depression. For instance, the mean positive overshoot shows that, on average, OOP health payments for households of persons with depression and high disability were 17.3% higher than the 10% of total consumption. At the same threshold, in households with depression and low disability, the percentage of households spending more than the threshold for health care was 8.1, and 7.6% in comparison households without depression. The corresponding values for the 25% threshold of total consumption were 21.5, 10.4 and 1.7% higher than the threshold for households containing a member with depression and high disability, with depression and low disability and without depression, respectively.

### Impact of out-of-pocket payments on poverty measures

Table 3 presents information on impoverishment. OOP health expenditure (post-payment head count) increased the percentage of poor households irrespective of the presence of a person with depression and disability in the household. Poverty increased by 5.8% in households with depression and high disability, by 3.5% in households with depression and low disability, and 2.3% in households without depression(*p* = 0.039).

**Table 3:** Impoverishing effect of out-of-pocket payments based on pre and post payments on health care

	Depression and high disability	Depression and low disability	Comparison without depression
Poverty head count			
Pre-payment head count ^A^	12.6%	12.3%	10.8%
Post payment head count ^B^	18.4%	15.8%	13.1%
Absolute percentage point change (impact) ^C^_(=B-A)_	**5.8%** ^***†***^	3.5%	2.3%
Relative percentage change (=C/A*100)	**46.0%** ^***†***^	28.4%	21.2%
Poverty gaps			
Prepayment poverty gap ^A^*$*	8.7	5.3	6.8
Post payment poverty gap ^B^*$*	9.9	5.6	6.9
Absolute point change (impact)^C^_(=B-A)_ *$*	**1.2** ^***†***^	0.3	0.1
Relative percentage change(=C/A*100)	**13.7%** ^***†***^	5.6%	1.5%
Normalized poverty gaps			
Pre-payment normalized gap ^A^	4.7%	2.9%	3.7%
Post-payment normalized gap ^B^	5.4%	3.0%	3.8%
Absolute percentage point change (impact) ^C^_(=B-A)_	0.7%	0.1%	0.1%
Relative percentage change(=C/A*100)	**14.8%** ^***†***^	3.4%	2.7%

$ (USD), USD1 = Birr 20.69 (2015);

Bold, significant at P < 0.05 for Pearson’s χ^2^; ^**†**^ for Kruskal-Wallis comparing pre and post payment by depression sub-groups

The poverty gap increased from US$ 8.7 to US$ 9.9 for depression and high disability, US$ 5.3 to US$ 5.6 for depression and low disability, and US$ 6.8 to US$ 6.9 for control households without depression. This change in poverty gap following out-of-pocket payments was significantly higher(*p* = 0.047) for households having a member with depression and high disability.

### Factors associated with catastrophic out-of-pocket payments

The incidence of catastrophic payment accounting for differences in socio-demographic and economic status is shown in Table 4. The risk of catastrophic OOP payments was significantly related to the level of disability. Households of persons with depression and high disability were three times more likely to experience catastrophic OOP payments than households without persons with depression (RR:2.1, 95%CI: 1.1, 4.6). Likewise, urban households (RR:1.6; 95%CI: 1.0, 3.3) were significantly more likely to report catastrophic OOP payments. In contrast, households with no children were less likely to experience catastrophic payments (RR: 0.2; 95% CI: 0.06, 0.8).

**Table 4:** Predictors of catastrophic health expenditure among households of a person with depression and comparison households

Factors	Catastrophic headcount*§* N (%)	Unadjusted model	Adjusted model
		RR (95%CI)	RR (95%CI)
Mental health condition			
Depression and high disability	13 (24.0)	**1.9 (1.0–4.1)**	**2.1 (1.1–4.6)**
Depression and low disability	8 (15.3)	1.2 (0.4–2.9)	1.3 (0.5–3.1)
Comparison without depression	10 (12.2)	1.00^*†*^	1.00^*†*^
Area of residence			
Urban	8 (21.6)	**1.4 (1.0–2.9)**	**1.6 (1.0–3.3)**
Rural	23 (15.2)	1.00^*†*^	1.00^*†*^
Gender of the household head			
Male	27 (17.2)	1.2 (0.5–2.6)	0.9 (0.3–2.1)
Female	6 (14.6)	1.00^*†*^	1.00^*†*^
Consumption quintile			
Quintile 1 (lowest)	6 (21.4)	1.5 (0.5–4.1)	1.1 (0.4–3.1)
Quintile 2	2 (6.6)	0.4 (0.1–2.1)	0.3 (0.08–1.5)
Quintile 3	7 (18.4)	1.3 (0.5–3.5)	0.8 (0.3–2.2)
Quintile 4	9 (21.9)	1.5 (0.6–3.9)	1.1 (0.4–2.6)
Quintile 5 (highest)	7 (13.7)	1.00^*†*^	1.00^*†*^
Children in the household			
0	2 (7.4)	**0.3 (0.05–0.9)**	**0.2 (0.06–0.8)**
1 or 2	13 (15.4)	0.7 (0.3–1.4)	0.5 (0.2–1.2)
3 or more	16 (20.7)	1.00^*†*^	1.00^*†*^
Household head education			
No formal education	19 (17.4)	0.9 (0.3–2.4)	0.8 (0.3–2.2)
Primary education	7 (14.0)	0.7 (0.2–2.2)	0.5 (0.2–1.6)
More than primary education	5 (17.8)	1.00^*†*^	1.00^*†*^
Household having a member above 60 years old			
Yes	26 (17.4)	1.2 (0.5–3.1)	1.0 (0.4–2.5)
No	5 (13.5)	1.00^*†*^	1.00^*†*^

§Catastrophic defined as health payments ≥ 10% of total consumption. †; reference group

CI, confidence interval; RR, risk ratio; Bold, significant at P < 0.05

### Financial coping strategies

Higher proportions of households with depression and high disability implemented various coping strategies compared with households without depression. For example, drawing up accounts at shops (34.6% vs. 28.0%), taking a loan from a bank or financial institution (28.8% vs.18.2%),reducing food consumption (36.5%vs.23.1%), reducing medical visits (36.5%vs.8.5%) and withdrawing children from school (15.3% vs. 6.1%).See Table 5.

**Table 5:** Financial coping strategies adopted by households with and without a member living with depression

Coping strategies	Depression and high disability (*n* = 52)		Depression and low disability (*n* = 54)		Comparison group without depression (*n* = 82)	
	No	% (95% CI)	No	% (95% CI)	No	% (95% CI)
Drew up accounts at shops	18	34.6 (21.2–47.9)	12	22.2 (10.7–33.6)	23	28.0 (18.1–37.9)
Loan from Bank or financial institution	15	28.8 (16.1–41.5)	8	14.8 (5.0–24.6)	15	18.2 (9.7–26.8)
Reduced food consumption	19	36.5 (23.0–50.0)	15	27.7 (15.4–40.1)	19	23.1 (13.8–32.4)
Reduced medical visits	19	36.5 (23.0–50.0)	12	22.2 (10.7–33.6)	7	8.5 (2.3–14.7)
Received support from relatives	18	34.6 (21.2–47.9)	14	25.9 (13.8–37.9)	19	23.1 (13.8–32.4)
Withdrew children from school	8	15.3 (5.2–.25.5)	6	11.1 (2.4–19.7)	5	6.1 (0.8–11.3)
Took on paid extra work	17	32.6 (19.5–45.8)	14	25.9 (13.8–37.9)	26	31.7 (21.4–41.9)
Used savings	4	7.6 (0.2–15.1)	9	16.6 (6.3–26.9)	10	12.2 (4.9–19.4)
Sold assets	37	74.0 (61.4–86.5)	48	88.8 (80.2–97.5)	60	78.9 (69.5–88.3)

Table 6 shows the results from multivariate analysis of the relative risk of financial coping strategies among households with and without depression. Households of persons with depression and low disability were at significantly higher risk (RR: 1.1; 95% CI: 1.1, 1.3) of selling assets to cope with financial difficulties compared with households without a member with depression. In households of persons with depression and high disability (RR:4.4; 95% CI:2.1, 9.3) and households of persons with depression and low disability (RR: 2.3; 95%CI: 1.0,5.7),the risk of reducing medical visits was significantly higher than among households without a person with depression. Households having a member with depression and high disability were at significantly higher risk of cutting food consumption for financial coping (RR:2.0; 95%CI: 1.1, 4.5) compared with households without a member with depression. Withdrawing children from school was about three times higher for households with depression and high disability compared with households without depression (RR: 3.0; 95%CI: 1.0, 8.5).

**Table 6:** Un- adjusted and adjusted risk ratios (RR) for coping strategies for financial constraints by households with and without a member living with depression

Characteristics	Coping strategies implemented for financial constraints							
	Sold assets	Drew up accounts at shops	Cut down food consumption	Withdrew children from school	Supported by relatives	Reduced medical visits	Used savings	Took on extra work
A) Unadjusted model								
	RR (95% CI)	RR (95%CI)	RR (95%CI)	RR (95%CI)	RR (95% CI)	RR (95% CI)	RR (95% CI)	RR (95% CI)
Depression and high disability	0. 9 (0.7–1.1)	1.2 (0.7–2.0)	**1.6 (1.0–2.7)**	**2.5 (1.1–7.3)**	1.5 (0.8–2.6)	**4.2 (1.9–9.4)**	0.6 (0.2–1.9)	1.0 (0.6–1.7)
Depression and low disability	1.1 (0.9–1.3)	0.7 (0.4–1.4)	1.2 (0.6–2.1)	1.8 (0.5–5.6)	1.1 (0.6–2.0)	**2.6 (1.0–6.1)**	1.3 (0.6–3.1)	0.8 (0.4–1.4)
Comparison group without depression	1.00^†^	1.00^†^	1.00^†^	1.00^†^	1.00^†^	1.00^†^	1.00^†^	1.00^†^
b) Adjusted model ^#^								
	RR (95% CI)	RR (95%CI)	RR (95%CI)	RR (95%CI)	RR (95% CI)	RR (95% CI)	RR (95% CI)	RR (95% CI)
Depression and high disability	0.9 (0.7–1.1)	1.1 (0.4–2.5)	**2.0 (1.1–4.5)**	**3.0 (1.0–8.5)**	1.5 (0.9–2.6)	**4.4 (2.1–9.3)**	0.4 (0.1–1.5)	0.8 (0.5–1.3)
Depression and low disability	**1.1 (1.0–1.3)**	0.7 (0.4–1.4)	1.1 (0.5–2.7)	1.5 (0.4–5.1)	1.0 (0.5–2.0)	**2.3 (1.0–5.7)**	1.3 (0.6–3.1)	1.1 (0.8–1.5)
Comparison group without depression	1.00^†^	1.00^†^	1.00^†^	1.00^†^	1.00^†^	1.00^†^	1.00^†^	1.00^†^

^#^ The model included control variables (i.e. residence, gender and consumption quintiles); CI, confidence interval; RR, risk ratio; †; reference group

Bold, significant at P < 0.05

## Discussion

Ethiopia has taken steps towards health care financing reform in addressing the universal health coverage components of financial protection [42]. However, progress has been slow, and many health facilities continue to rely on user fees. There has been limited focus on the financial burden borne by households due to out-of-pocket healthcare costs, including for people with mental health conditions.

According to the results of this study, households of persons with depression and high disability had a higher incidence of catastrophic expenditure irrespective of threshold levels. At the 10% threshold, about one in every four households with high disability depression reported catastrophic OOP payments in the preceding one month.

The higher incidence of catastrophic payments in households of persons with depression and high disability may result from the tendency for people with depression to present repeatedly to health services with somatic complaints and receive non-specific treatments which do not address the underlying problem. This leads to further help-seeking and expenditure on investigations and medications, which may account for higher OOP payments. Previous studies from Ethiopia reported similar findings [18, 19]. A higher prevalence of disabilities is known to be associated with increased OOP health payments in LMICs [43]. In a study in Tanzania, functional disability led to increased OOP health expenditures [44]. Nonetheless, our finding of 24.0% catastrophic OOP payments for households of persons with depression and high disability at the 10% threshold is higher than the 14.6% reported in an earlier study from India [16]. The higher rate in our study may be attributable to the predominantly rural, low-income setting of the current study while the Indian study was conducted in a more affluent area, with high literacy. Furthermore, India has different health care financing and financial protection mechanisms.

At a 25% threshold of household total consumption expenditure, our findings of 5.7% of catastrophic costs with depression and high disability and 5.5% with depression and low disability are relatively higher than the multi-country report of 2.6% [10]. A plausible explanation is that depression is very costly/burdensome. Moussavi et al. (2007) compares disability from depression to other chronic conditions and finds that depression is always more burdensome [45]. Thus, persons with depression and disability face higher financial risk due to the need for treatment and care.

Our findings highlight that the burden of OOP payments on households with a person with depression is heavy. The mean positive overshoot of catastrophic OOP payments at the 10% threshold shows that, on average, catastrophic expenditure was higher by 17.3% for households with depression and high disability compared with 7.6% for households of persons without depression. This translates into households with depression and high disability spending 27.3% of their total expenditure on health compared to 17.6% for households without depression. Our results relating to mean positive overshoot for households with persons with depression and high disability appear to be higher than the 24%reported by Tolla et al. (2016) for Ethiopia on OOP costs for cardiovascular diseases in cardiac hospitals [46]. This indicates that households with depression and high disability are, in most cases, exposed to a greater risk of catastrophic health spending.

Our analysis demonstrated that catastrophic OOP payments were more prevalent in urban residents and in the poorest households. A previous study in Ethiopia reported a similar finding [47]. This might be because urban residents visit health clinics more often. Urban residents may also be more likely to visit private health facilities that charge significantly higher fees. The higher proportion of catastrophic OOP payments among the poorest shows that OOP payments are regressive and that there is a lack of financial risk protection for poor households against illness. This is explained by the low number (3%) of households enrolled in the government’s financial protection scheme. This study has demonstrated that households that have no children had a lower risk of incurring catastrophic OOP payments compared with households having children. The reason may be that children are more likely to get ill and incur medical costs. This finding is in line with earlier studies conducted in low -and middle-income countries [48, 49]. Our study findings also reinforce the well documented relationship between poverty and mental illness [50]. We found prepayment poverty headcount to be more prevalent, about 2% points higher in households with depression and high disability compared with households without depression.

Both poverty headcount and poverty gap become higher after OOP payments for health care. We found that 5.8% of households of persons with depression and high disability, 3.5% with depression and low disability, and 2.3% of households without depression, fell into poverty due to OOP payments for healthcare. Although it is not always possible to directly compare findings due to methodological differences, this finding is higher than that reported for the African region (1.4% in 2010) [15]. The difference might be due to the poverty line measurement used: Wagstaff and colleagues used the 2011 $1.90-a-day poverty line while in our study we used the national poverty line (US$0.497-a-day). We argue that estimating poverty impact using a locally defined poverty line is more appropriate as the local price levels of household consumables are thereby considered.

The average shortfall from the poverty line (poverty gap) following OOP payments was substantial. On average, OOP increased the poverty gap by 13.7% in households with depression and high disability, by 5.6% in households with depression and low disability and by 1.5% in household without depression. These findings highlight that the pre-payment poor became even poorer and some who were not poor before payment became so after paying for health care.

The risk of adopting financial coping strategies varied by the presence of a person with depression and with the level of disability. We found that depression and higher disability increased the risk of interrupting medical visits. Other studies have also reported that non-adherence to prescribed medications and loss to follow-up is common in the treatment of depression [21, 51]. In a recent study from Sodo, Ethiopia, drop-out from care was reported to be mostly due to poverty in people with severe mental disorder [52]. We argue that non-adherence to treatment minimizes health care payments (i.e. OOP costs were relatively low because of low use of hospital care) and underestimates the incidence of catastrophic expenditure. Furthermore, as reported elsewhere, such practices will have a negative consequence on outcomes of the condition [51]. Our finding on selling assets by households of persons with depression and low disability for financial constraint is similar to what was reported in other study [53].

This study also found that depression and high disability significantly increased the risk of withdrawing children from school. This is consistent with a previous study that reported school dropout and absenteeism as being associated with maternal common mental disorders, mostly comprising depression and anxiety [54]. Withdrawing children from school might be for intra-household labour substitution, whereby in resource-poor settings children are obliged to take on the work activities of a sick parent. Similarly, children are also involved in providing care for the sick household member. However, school withdrawal may have broader livelihood impacts on future generations, leading to inter-generational transmission of poverty, a key target of the Sustainable Development Goals. In line with this, in our previous study in the same population, we found similar findings in households of persons with severe mental disorders such as psychosis and bipolar disorder [55].

Our study findings support the robust evidence base regarding the association between illness and reduction in food consumption through pathways of medical expenditure [56]. Indeed, in the situation of illness, financial constraints may trigger reduction of food consumption which will result in household food insecurity. In South Africa, maternal depression was associated with household food insecurity [57]. In Ethiopia, Hadley et al. (2008) reported depression was independently associated with food insecurity [58]. In India, a common response by households to financial difficulties during illness was to change consumption patterns in order to cover health care costs [59].

The strength of our study is that, to our knowledge, this is the first study to examine catastrophic OOP payments and impoverishment in households of a person with and without depression and associated financial coping strategies from any sub-Saharan African country. We estimated expenditures in a comprehensive and systematic way and analyzed poverty using a local poverty line. The inclusion of a comparison group without depression in our study enabled us to estimate the net effect of depression and associated disability on out-of-pocket health expenditures.

Nevertheless, our study is not without limitations. The study samples were drawn from health facilities which may not be generalizable to the general population. The cross-sectional design of the study precludes any interpretation of causality. Our comparison households for depression may have included a person with depression, as we did not screen all members of the household. Information on transportation costs was not included in the estimates although such costs represent a substantial economic burden. We did not assess for co-morbid physical and mental health conditions in households in our samples. Our sample size was relatively small, meaning that we may have been under-powered to detect moderate effects. In view of the small sample size and methodological challenges, our findings can at most be taken as indicative of the burden of OOP payments on households. Moreover, the study should be seen in light of being one of the very first attempts at assessing catastrophic health expenditure and impoverishment in households of persons with depression in LMIC. Multiple hypothesis testing could have led to spurious findings; however, the pattern of associations across outcomes strengthens confidence in the findings.

## Conclusions

Our study shows that households having a member with depression and high disability faced disproportionately higher catastrophic OOP payments and impoverishment. Factors such as residential location and the presence of children in the household influenced catastrophic OOP health payments. Depression and high disability increased the risk of interrupting medical visits, cutting down food consumption and withdrawing children from school. Financial protection interventions, including prepayment schemes, exemptions and fee waiver strategies, need to target households of persons with depression.

## Additional file

Additional file 1: **Table S1.** Expenditure categories for consumption by depression group. (DOCX 13 kb)

## Data Availability

The data are being used for a PhD student (YH) for his thesis and are not, therefore, available at the present time to the general public. The data may be requested from the corresponding author for verification of the analyses in this paper on a reasonable request.

## Abbreviations

Emerald: Emerging mental health systems in low- and middle-income countries
GDP: Gross domestic product
LMICs: Low- and middle-income countries
OECD: Organization of economic co-operation and development
OOP: Out-of-pocket
PHQ-9: Patient health questionnaire – 9 item
PRIME: Programme for improving mental health care
SAGE: Study on global Ageing and adult health
SDGs: Sustainable development goals
UHC: Universal health coverage
WHO: World health organization
WHODAS: WHO disability assessment schedule

## References

[1] World Health Organization (2001). World Health Report 2000. Health systems: improving performance.

[2] World Health Organization and the International Bank for Reconstruction and Development / The World Bank. Tracking universal health coverage: 2017 global monitoring report. WHO and the World Bank. 2017. http://www.who.int/healthinfo/universal_health_coverage/report/2017/en. Accessed 11 Mar 2018.

[3] Federal Democratic Republic of Ethiopia. Ministry of Health. Ethiopia Sixth Health Accounts. 2013/14. Addis Ababa. Ethiopia. 2017. http://apps.who.int/nha/database/DocumentationCentre/Index/en. Accessed 12 Mar 2018.

[4] Purvis G, Alebachew A, Feleke W. Ethiopia health sector financing reform midterm project evaluation. Washington, DC: Global Health Technical Assistance Project; 2011. http://dec.usaid.gov. Accessed 15 Mar 2018.

[5] Russell S, Abdella K (2002). Too poor to be sick: coping with the costs of illness in east Hararghe, Ethiopia.

[6] Bogale T, Haile Mariam D, Ali A (2005). Costs of illness and coping strategies in a coffee-growing rural district of Ethiopia. J Health PopulNutr.

[7] Whitehead M, Dahlgren G, Evans T (2001). Equity and health sector reforms: can low-income countries escape the medical poverty trap?. Lancet..

[8] Berki SE (1986). A look at catastrophic medical expenses and the poor. Health Aff.

[9] Ranson MK (2002). Reduction of catastrophic health care expenditures by a community-based health insurance scheme in Gujarat, India: current experiences and challenges. Bull World Health Organ.

[10] Wagstaff Adam, Flores Gabriela, Hsu Justine, Smitz Marc-François, Chepynoga Kateryna, Buisman Leander R, van Wilgenburg Kim, Eozenou Patrick (2018). Progress on catastrophic health spending in 133 countries: a retrospective observational study. The Lancet Global Health.

[11] Xu K (2005). Distribution of health payments and catastrophic expenditures methodology. Discussion paper, department ‘health system financing’ (HSF).

[12] Russell S, Gilson L (2006). Are health services protecting the livelihoods of the urban poor in Sri Lanka? Findings from two low-income areas of Colombo. SocSci Med.

[13] Murray CJ, Xu K, Klavus J, Kawabata K, Hanvoravongchai P, Zeramdini R, Aguilar-Rivera AM, Evans DB, Murray CJL, Evans DB (2005). Assessing the distribution of household financial contributions to the health system: concepts and empirical application. Health Systems Performance Assessment: Debates, Methods and Empiricism.

[14] Wagstaff A, van Doorslaer E (2003). Catastrophe and impoverishment in paying for health care: with applications to Vietnam 1993-1998. Health Econ.

[15] Wagstaff Adam, Flores Gabriela, Smitz Marc-François, Hsu Justine, Chepynoga Kateryna, Eozenou Patrick (2018). Progress on impoverishing health spending in 122 countries: a retrospective observational study. The Lancet Global Health.

[16] Patel V, Chisholm D, Kirkwood BR, Mabey D (2007). Prioritizing health problems in women in developing countries: comparing the financial burden of reproductive tract infections, anaemia and depressive disorders in a community survey in India. Tropical Med Int Health.

[17] Chisholm D, Sekar K, Kumar KK, Saeed K, James S, Mubbashar M (2000). Integration of mental health care into primary care. Demonstration cost-outcome study in India and Pakistan. Br J Psychiatry.

[18] Mogga S, Prince M, Alem A, Kebede D, Stewart R, Glozier N, Hotopf M (2006). Outcome of major depression in Ethiopia: population-based study. Br J Psychiatry.

[19] Hanlon C, Medhin G, Selamu M, Breuer E, Worku B, Hailemariam M (2015). Validity of brief screening questionnaires to detect depression in primary care in Ethiopia. J Affect Disord.

[20] Wright DR, Katon WJ, Ludman E, McCauley E, Oliver M, Lindenbaum J, Richardson LP (2016). The Association of Adolescent Depressive Symptoms with healthcare utilization and payer-incurred expenditures. AcadPediatr..

[21] Fekadu A, Medhin G, Selamu M (2017). W. Giorgis T, Lund C, Alem a, Prince M, Hanlon C. recognition of depression by primary care clinicians in rural Ethiopia. BMC Fam Pract.

[22] Fischer Michael, Ramaswamy Rohit, Fischer-Flores Luz, Mugisha Grace (2018). Measuring and Understanding Depression in Women in Kisoro, Uganda. Culture, Medicine, and Psychiatry.

[23] Garland AF, Deyessa N, Desta M, Alem A, Zerihun T, Hall KG, Goren N, Fish I (2018). Use of the WHO's perceived well-being index (WHO-5) as an efficient and potentially valid screen for depression in a low income country. Fam Syst Health.

[24] American Psychiatric Association (2013). Diagnostic and statistical manual of mental disorders, 5th edition: DSM-5.

[25] World Health Organization (1992). The ICD-10 Classification of Mental and Behavioural Disorders.

[26] World Health Organization (2016). Mental Health Gap Action Programme Intervention Guide (mhGAP-IG) for mental, neurological and substance use disorders in non-specialized health settings. version 2.0.

[27] Semrau M, Evans-Lacko S, Alem A, Ayuso-Mateos JL, Chisholm D, Gureje O, Hanlon C, Jordans M, Kigozi F, Lempp H, Lund C, Petersen I, Shidhaye R, Thornicroft G (2015). Strengthening mental health systems in low- and middle-income countries: the Emerald programme. BMC Med.

[28] Hanlon C, Luitel N, Kathree T, Murhar V, Shrivasta S, Medhin G, Ssebunnya J, Fekadu A, Petersen I, Jordans M, Kigozi F, Thornicroft G, Patel V, Tomlinson M, Lund C, Breuer E, De Silva M, Prince M (2014). Challenges and opportunities for implementing mental health in primary care in the PRIME study: comparison of the baseline situation in five LAMIC settings. PLoS One.

[29] Lund C, Tomlinson M, De Silva M, Fekadu A, Shidhaye R, Jordans M, Petersen I, Bhana A, Kigozi F, Prince M, Thornicroft G, Hanlon C, Kakuma R, McDaid D, Saxena S, Chisholm D, Raja S, Kippen-Wood S, Honikman S, Fairall L, Patel V (2012). PRIME: a Programme to reduce the treatment gap for mental disorders in five low- and middle-income countries. PLoS Med.

[30] Fekadu A, Hanlon C, Medhin G, Alem A, Selamu MW, Giorgis T, Shibre T, Teferra S, Tegegn T, Breuer E, Patel V, Tomlinson M, Thornicroft G, Prince M, Lund C (2016). Development of a scalable mental healthcare plan for a rural district in Ethiopia. Br J Psychiatry.

[31] Kroenke K, Spitzer RL, Williams JB (2001). The PHQ-9: validity of a brief depression severity measure. J Gen Intern Med.

[32] Gelaye B, Williams MA, Lemma S, Deyessa N, Bahretibeb Y, Shibre T, Wondimagegn D, Lemenhe A, Fann JR, Van der Stoep A, Zhou X-HA (2013). Validity of the patient health questionnaire-9 for depression screening and diagnosis in East Africa. Psychiatry Res.

[33] World Health Organization (2013). World Health Organization study on global ageing and adult health (SAGE).

[34] O’Donnell O, van Doorslaer E, Wagstaff A, Lindelow M (2008). Analyzing health equity using household survey data. A guide to techniques and their implementation.

[35] Saito E, Gilmour S, Rahman MM, Gautam GS, Shrestha PK, Shibuya K (2014). Catastrophic household expenditure on health in Nepal: a cross-sectional survey. Bull World Health Organ.

[36] National Bank of Ethiopia. Annual report 2015/2016. http://www.nbe.gov.et/pdf/annualbulletin/Annual%20Report%202015-16%20N.pdf. Accessed 3 June 2017.

[37] EUROSTAT. Glossary: Equivalised disposable income. http://ec.europa.eu/eurostat/statistics-explained/index.php?title=Glossary:Equivalised_disposable_income&oldid=171112.

[38] Ministry of Finance and Economic Development (MoFED), Development Planning and Research Directorate. Ethiopia’s Progress towards Eradicating Poverty: An Interim Report on Poverty Analysis Study (2010/11). Addis Ababa: Ethiopia; 2012.

[39] World Health Organization (2010). Measuring Health and Disability: Manual for WHO Disability Assessment Schedule (WHODAS 2.0).

[40] Lauritsen JM, Bruus M (2004). EpiData (version 3.1). A comprehensive tool for validated entry and documentation of data.

[41] Stata Corp L.P. Stata/SE Version 13.1. Texas: USA; 2014.

[42] Alebachew A, Hatt L, Kukla M (2014). Monitoring and evaluating Progress towards universal health coverage in Ethiopia. PLoS Med.

[43] Sousa RM, Ferri CP, Acosta D, Albanese E, Guerra M, Huang Y, Jacob KS, Jotheeswaran AT, Rodriguez JJ, Pichardo GR, Rodriguez MC, Salas A, Sosa AL, Williams J, Zuniga T, Prince M (2009). Contribution of chronic diseases to disability in elderly people in countries with low and middle incomes: a10/66 dementia research group population-based survey. Lancet.

[44] Brinda EM, Andrés RA, Enemark U (2014). Correlates of out-of-pocket and catastrophic health expenditures in Tanzania: results from a national household survey. BMC International Health and Human Rights.

[45] Moussavi S, Chatterji S, Verdes E, Tandon A, Patel V, Ustun B (2007). Depression, chronic diseases, and decrements in health: results from the world health survey. Lancet..

[46] Tolla MT, Norheim OF, Verguet S, Bekele A, Amenu K, Abdisa SG, Johansson KA (2017). Out-ofpocket expenditures for prevention and treatment of cardiovascular disease in general and specialized cardiac hospitals in Addis Ababa, Ethiopia: a cross-sectional cohort study. BMJ Glob Health.

[47] Memirie ST, Metaferia ZS, Norheim OF, Levin CE, Verguet S, Johansson KA (2017). Household expenditures on pneumonia and diarrhoea treatment in Ethiopia: a facility-based study. BMJ Glob Health.

[48] Khan JM, Ahmed S, Evans TG (2017). Catastrophic healthcare expenditure and poverty related to out-of-pocket payments for healthcare in Bangladesh—an estimation of financial risk protection of universal health coverage. Health Policy Plan.

[49] Kimani DN, Mugo MG, Kioko UM (2016). Catastrophic health expenditures and impoverishment in Kenya. Eur Sci J.

[50] Lund C, De Silva M, Plagerson S, Cooper S, Chisholm D, Das J (2011). Poverty and mental disorders: breaking the cycle in low-income and middle-income countries. Lancet..

[51] Abegaz Tadesse Melaku, Sori Lamessa Melese, Toleha Hussien Nurahmed (2017). Self-Reported Adverse Drug Reactions, Medication Adherence, and Clinical Outcomes among Major Depressive Disorder Patients in Ethiopia: A Prospective Hospital Based Study. Psychiatry Journal.

[52] Hailemariam M, Fekadu A, Prince M, Hanlon C (2017). Engaging and staying engaged: a phenomenological study of barriers to equitable access to mental healthcare for people with severe mental disorders in a rural African setting. Int J Equity Health.

[53] Kruk ME, Goldmann E, Galea S (2009). Borrowing and selling to pay for health care in low- and middle-income countries. Health Aff.

[54] Mekonnen H, Medhin G, Tomlinson M, Alem A, Prince M, Hanlon C (2018). Impact of maternal common mental disorders on child educational outcomes at 7 and 9 years: a population-based cohort study in Ethiopia. BMJ Open.

[55] Hailemichael Y, Haile Mariam D, Tirfessa K, Docrat S, Alem A, Medhin G, Lund C, Chisholm D, Fekadu A, Hanlon C. Mental health problems and socioeconomic disadvantage: a controlled household study in rural Ethiopia. Int J Ment Health Syst. 2018;under review. 10.1186/s12939-019-1020-4 PMC6670213 31366362

[56] Chisholm D, Stanciole AE, Tan Torres ET, Evans DB (2010). Economic impact of disease and injury: counting what matters. Bmj..

[57] Abrahams Z, Lund C, Field S, Honikman S (2018). Factors associated with household food insecurity and depression in pregnant south African women from a low socio-economic setting a cross-sectional study. Soc Psychiatry Psychiatr Epidemiol.

[58] Hadley C, Tegegn A, Tessema F, Cowan JA, Asefa M, Galea S (2008). Food insecurity, stressful life events and symptoms of anxiety and depression in East Africa: evidence from the Gilgel gibe growth and development study. J Epidemiol Community Health.

[59] Kabir MA, Rahman A, Salway S, Pryer J (2000). Sickness among the urban poor a barrier to livelihood security. J Int Dev.

