# Supplementary material for: Catastrophic health expenditure and impoverishment in households of persons with depression: a cross-sectional, comparative study in rural Ethiopia
Source: BMC Public Health. 2019 Jul 11;19:930. doi: 10.1186/s12889-019-7239-6 (PMC6625021; doi:10.1186/s12889-019-7239-6)
Supplement: Supplementary file 1 — Table S1. Expenditure categories for consumption by depression group. (DOCX 13 kb) [file 12889_2019_7239_MOESM1_ESM.docx]

Supplementary table 1.Expenditure categories for consumption by depression group

| Expenditure categories | Households proportion of expenses on consumption items (95% CI) | | | |
| --- | --- | --- | --- | --- |
|  | Depression and high disability | Depression and low disability | Households without depression | Difference between groups, *P*^a^ |
| **% of total expenditure** | | | | |
| All food items | 74.9 (70.7- 79.2) | 76.5(72.7-80.4) | 78.7(75.9 - 81.4) | 0.209 |
| Household regular expenses | 14.7(11.5- 17.8) | 13.7(10.7-16.6) | 13.5(11.3-15.6) | 0.823 |
| Household big expenditure | 4.7 (3.2-6.3) | 4.5 (3.1-5.8) | 5.5 (3.8-7.2) | 0.777 |
| Total health expenditure | 7.5 (4.3-10.7) | 6.7(4.4 - 9.0) | 4.9 (3.7-6.2) | 0.238 |
| **% of health expenditure** | | | | |
| Consultation | 17.4 (10.2-24.6) | 20.0 (12.4-27.6) | 18.7(9.3-19.0) | 0.516 |
| Medicines | 71.0 (63.6- 78.4) | 71.2 (63.9- 78.4) | 72.0 (66.4-77.7) | 0.887 |
| Diagnostics | 24.7(15.7-33.7) | 30.0 (21.1-38.9) | 30.6 (23,1-38.2) | 0.478 |

*Food items (staple foods, vegetable, fruit, spices etc), regular household expenses (electricity, water, cooking, renting, clothing, transport, etc), Big expenditure include (education , durable goods ,cultural ceremony, entertainment, tax etc); health expenditure include expense on ( outpatient consultation, drugs, diagnostic , hospitalization, medical appliances, ambulance, etc)*; *CI, confidence interval ; Calculated using a χ*2 *tests.*
